# An epigenetic marker panel for recurrence risk prediction of low grade papillary urothelial cell carcinoma (LGPUCC) and its potential use for surveillance after transurethral resection using urine

**DOI:** 10.18632/oncotarget.2129

**Published:** 2014-06-23

**Authors:** Leonel Maldonado, Mariana Brait, Christina Michailidi, Enrico Munari, Tina Driscoll, Luciana Schultz, Trinity Bivalacqua, Mark Schoenberg, David Sidransky, George J Netto, Mohammad Obaidul Hoque

**Affiliations:** ^1^ Department of Otolaryngology-Head and Neck Surgery, Johns Hopkins School of Medicine, Johns Hopkins University, Baltimore, Maryland, USA; ^2^ Department of Pathology, Johns Hopkins University, Baltimore, Maryland, USA; ^3^ Department of Urology, Johns Hopkins University, Baltimore, Maryland, USA; ^4^ Department of Oncology, Johns Hopkins University, Baltimore, Maryland, USA; ^5^ Department of Gynecology and Obstetrics, Johns Hopkins University School of Medicine, Baltimore, Maryland, USA

**Keywords:** LGPUCC, Recurrence, Epigenetics, Biomarkers, DNA methylation

## Abstract

By a candidate gene approach, we analyzed the promoter methylation (PM) of 8 genes (*ARF, TIMP3, RAR-β2, NID2, CCNA1, AIM1, CALCA* and *CCND2*) by quantitative methylation specific PCR (QMSP) in the DNA of 17 non-recurrent and 19 recurrent noninvasive low grade papillary urothelial cell carcinoma (LGPUCC) archival tissues. Among the genes tested, by establishing an empiric cutoff value, *CCND2, CCNA1, NID2*, and *CALCA* showed higher frequency of methylation in recurrent than in non-recurrent LGPUCC: *CCND2* 10/19 (53%) vs. 2/17 (12%) (p=0.014); *CCNA1* 11/19 (58%) vs. 4/17 (23.5%) (p=0.048); *NID2* 13/19 (68%) vs. 3/17 (18%) (p=0.003) and *CALCA* 10/19 (53%) vs. 4/17 (23.5%) (p=0.097), respectively. We further analyzed PM of *CCND2, CCNA1*, and *CALCA* in urine DNA from UCC patients including LGPUCC and controls. The frequency of *CCND2, CCNA1*, and *CALCA* was significantly higher (p<0.0001) in urine of UCC cases [38/148 (26%), 50/73 (68%) and 94/148 (63.5%) respectively] than controls [0/56 (0%), 10/60 (17%) and 16/56 (28.5%), respectively)]. Most importantly we found at least one of the 3 markers were methylated positive in 25 out of 30 (83%) cytology negative LGPUCC cases. We also explored the biological function of *CCNA1* in UCC. Prospective confirmatory studies are needed to develop a reliable tool for prediction of recurrence using primary LGPUCC tissues and/or urine.

## INTRODUCTION

In 2014, approximately 74,690 new cases will be diagnosed with bladder cancer and about 15,580 people will die from this disease in the United States [1]. Although men are diagnosed with bladder cancer at nearly three times the rate of women, women present with more advanced disease [2].

Urothelial cell carcinoma (UCC) constitutes over 90% of bladder cancers in the Western world. Non-muscle invasive UCC is the most common at presentation (around 75%) and is treated by trans-urethral resection of bladder tumor (TURBT) with or without BCG where 20% of patients will be cured, 70% will recur at least once every 5 years, and the remaining will progress to muscle-invasive disease with poor prognosis [3]. Currently, there are no well validated markers that can discern the tumors that will recur from those that will not. Moreover, conventional approaches (computed tomography, urine cytology, histopathology, or tumor-node-metastasis classification) are not ideal to predict risk of recurrence. Hence, it is crucial to develop molecular markers that can predict recurrence at the time of diagnosis, and that such markers would allow a more individualized therapy, and overall management based on a patient's risk. Furthermore, it would also be important to develop a test that could provide cost-effective, non-invasive monitoring for low-risk patients, while using a more active approach to identify high-risk cancers before they progress [4]. Numerous potential markers have been proposed, such as Ki-67, TP53 and TERT, which have some promising correlation, but no conclusive evidence has been shown [4, 5].

Cancer is a genetic disease, and in some cancers such as UCC, environmental factors play an important role in cancer initiation. Accumulated evidence over the last two decades also suggest that epigenetic changes play an essential role in carcinogenesis and contribute to the development and progression of tumor cells [6]. They include DNA methylation, histone modifications, and nucleosome repositioning [7, 8]. DNA methylation is defined as the addition of a methyl group on a cytosine that precedes a guanosine (known as CpG). There are CpG-rich regions known as CpG islands, which usually span the 5'end region of many genes with tumor suppressor activity and are usually unmethylated in normal cells [9]. Promoter methylation is a common mechanism for gene inactivation [6, 7, 10] and has been found to be a potential biomarker for several types of cancer, including UCC [11, 12].

In the current study, by a candidate gene approach, we selected 8 genes (*ARF, TIMP3, RAR-β2, NID2, CCNA1, AIM1, CALCA*, and *CCND2*) that had been previously shown to be frequently methylated in UCC by our group and others [11-14], and based on their reported functional characteristics. Briefly, cyclins belong to a highly conserved family, and the members are characterized by a dramatic periodicity in protein abundance through the cell cycle. Cyclins function as regulators of CDK kinases. Different cyclins exhibit distinct expression and degradation patterns which contribute to the temporal coordination of each mitotic event [15]. We previously reported that *CCNA1* is frequently methylated in solid tumors including UCC [11, 16]. Functionally, CCND2 plays different roles in different cancer types. While silencing of *CCND2* expression by promoter methylation is associated with cancer progression in some cancer types [17-20], over-expression of *cyclin D2* correlates with progression and poor prognosis in other tumor types [21-24]. We selected *CCND2* based on our previous findings in UCC [11]. Similarly, the remaining genes were selected based on their relationship with cell growth and known cancer specific methylation in different solid tumors including UCC [11-14, 25, 26] (further explored in the discussion section).

In this study, we analyzed 8 genes (*ARF, TIMP3, RAR-β2, NID2, CCNA1, AIM1, CALCA* and *CCND2*) in a group of retrospectively collected 36 low-grade papillary urothelial cell carcinoma (LGPUCC) patients with recurrent and non-recurrent tumors. As methylation of *CCNA1* showed significant correlation with recurrence, we further explored its biological function in UCC cell lines *in vitro*. Finally, we evaluated the feasibility of detecting UCC (including LGPUCC) in bodily fluids by analyzing promoter methylation of a panel of three genes in urine samples obtained from controls (subjects without any known cancer) and UCC patients; these three genes had not been tested in urine samples before for detection of LGPUCC.

## RESULTS

Our study was divided into three parts: 1) To determine whether any of the candidate methylated genes or panel of genes has the potential to predict recurrence by testing primary tumors; 2) To test a panel of candidate genes that were found to be related to recurrence in primary tissue analysis and to determine the potential of these methylated genes for non-invasive detection of LGPUCC in urine; 3) To evaluate the functional significance of promoter methylation and silencing of *CCNA1* in UCC cell lines.

### Methylation frequency of primary recurrent and non-recurrent UCC

**Figure 1:**
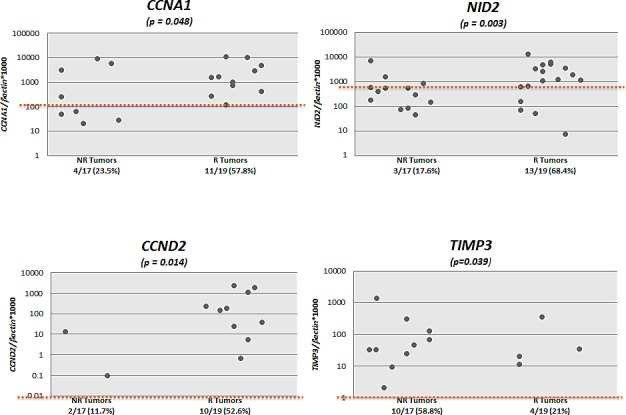

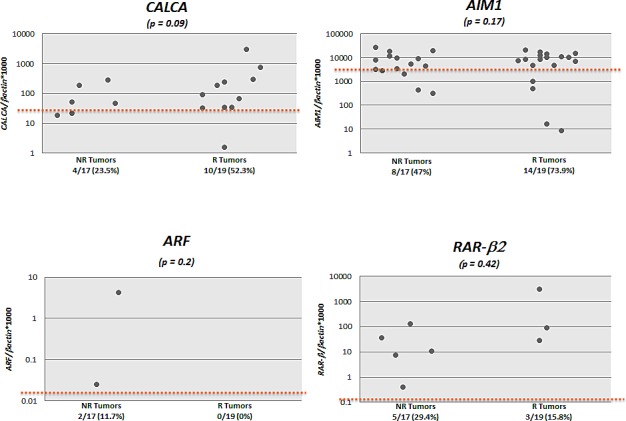
Scatter plots of quantitative methylation values of all the 8 genes tested in recurrent (R, n=19) and non-recurrent (NR, n=17) primary urothelial cell carcinoma (UCC) samples Calculation of the gene of interest/β-*actin* ratios was based on the fluorescence emission intensity values for both the gene of interest and β-*actin* obtained by quantitative real-time PCR analysis. The obtained ratios were multiplied by 1,000 for easier tabulation. Zero values cannot be plotted correctly on a log scale.

We tested the promoter methylation of 8 genes (*ARF, TIMP3, RAR- β2, NID2, CCNA1, AIM1, CALCA* and *CCND2*) in DNA from primary non-recurrent and recurrent LGPUCC tissues. By establishing empiric cutoff values, *CCND2, CCNA1, NID2*, and *CALCA* showed a significantly higher frequency of methylation in recurrent than in non-recurrent LGPUCC (Table 2A). The methylation frequency of an individual gene in recurrent and non-recurrent LGPUCC respectively was: *CCND2* 10/19 (52.6%) vs. 2/17 (11.7%) (p=0.014); *CCNA1* 11/19 (57.9%) vs. 4/17 (23.5%) (p=0.048); *NID2* 13/19 (68.4%) vs. 3/17 (17.6%) (p=0.003); and *CALCA* 10/19 (52.6%) vs. 4/17 (23.5%) (p=0.097). Scatter plots of all the 8 genes tested are shown in Figure 1.

**Table 1:** Demographic and clinicopathological data of primary LGUCC samples*

	
Age at diagnosis (years)	
Median	66.4
Range	31-89
Recurrence	
Recurrent	19 (52.7%)
Non-recurrent	17 (47.2%)
Race	
Caucasian	31 (86.1%)
African-american	2 (5.6%)
Unknown	3 (8.3%)
Gender	
Male	30 (83%)
Female	6 (17%)
Smoking	
Smoker	22 (61.1%)
Non-smoker	10 (27.8%)
Unknown	4 (11.1%)

* All patients were diagnosed with Low Grade Papillary Urothelial Cell Carcinoma

Table 2Promoter methylation frequency in tissues and urines

**A:** Promoter methylation frequency for the 8 genes analyzed in the primary LGPUCC samples (non-recurrent versus recurrent)

GENE	Methylation positive% (number of methylation positive/number of total cases)		Fisher's exact test p-value
	Non-recurrent tumors	Recurrent tumors	
*CCND2*	2/17 (11.7%)	10/19 (52.6%)	0.014*
*CCNA1*	4/17 (23.5%)	11/19 (57.9%)	0.048*
*CALCA*	4/17 (23.5%)	10/19 (52.6%)	0.097
*AIM1*	8/17 (47%)	14/19 (73.9%)	0.171
*NID2*	3/17 (17.6%)	13/19 (68.4%)	0.003*
*ARF*	2/17 (11.7%)	0/19 (0%)	0.216
*TIMP3*	10/17 (58.8%)	4/19 (21%)	0.039*
*RARβ2*	5/17 (29.4%)	3/19 (15.8%)	0.434

* p values <0.05 were considered statistically significant

**B:** Promoter methylation of *CCND2, CCNA1* and *CALCA* in urine of UCC patients and controls, and its association with clinicopathological parameters

	I. Promoter methylation frequency in urine from controls and UCC cases		
GENE	Methylation positive% (number of methylation positive/number of total cases)		Fisher's exact test p-value
	Normal urines (controls)	UCC urines	
*CCND2*	0/56 (0%)	38/148 (25.6%)	<0.0001*
*CCNA1*	10/60 (16.6%)	50/73 (68.4%)	<0.0001*
*CALCA*	16/56 (28.5%)	94/148 (63.5%)	<0.0001*
			
	II. Association of Promoter methylation determined in urine with grade and stage of UCC		
GENE	Methylation positive% (number of methylation positive/number of total cases)		Fisher's exact test p-value
	LGUCC	HGUCC	
*CCND2*	35/101 (34.6%)	3/24 (12.5%)	0.047*
*CCNA1*	35/52 (67.3%)	7/14 (50%)	0.348
*CALCA*	76/101 (75.2%)	8/24 (33.3%)	0.0002*
	Non-invasive stage (Stage 1)	Invasive stages (Stage 2, 3)	
*CCND2*	3/32 (9.3%)	35/92 (38.1%)	0.002*
*CCNA1*	9/16 (56.3%)	34/49 (69.4%)	0.372
*CALCA*	17/32 (53.1%)	67/92 (72.8%)	0.049*

* p values <0.05 were considered statistically significant

### Methylation frequency of a panel of genes in the urine of UCC patients and controls

**Figure 2:**
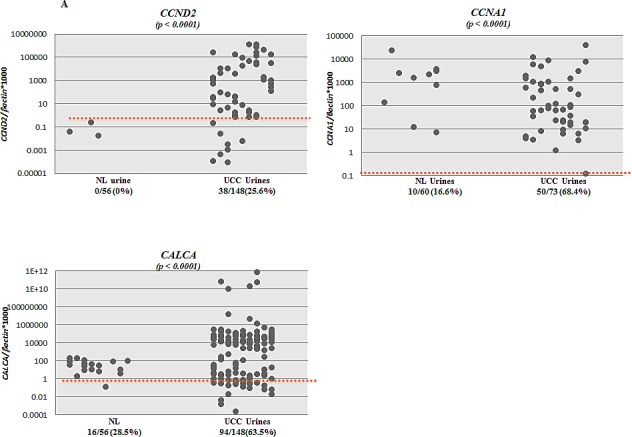

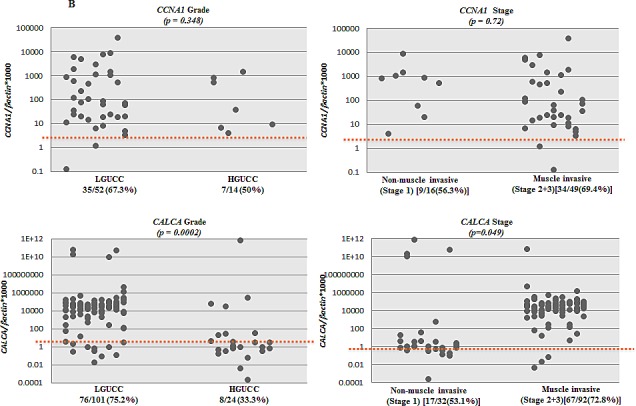

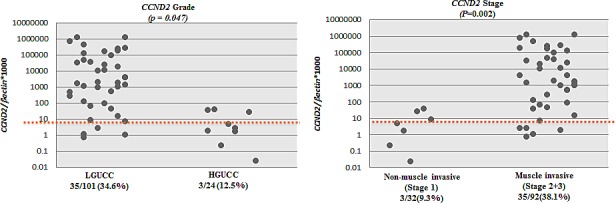
Scatter plots showing the extent of methylation in *CCNA1, CCND2* and *CALCA* genes in urine sediments; **A**. Methylation levels of *CCNA1, CCND2* and *CALCA* genes in urine sediment DNA of UCC patients (148 for *CCND2*, 73 for *CCNA1* and 148 for *CALCA*) and no known neoplastic disease subjects (56 for *CCND2*, 60 for *CCNA1* and 56 for *CALCA*). NL=Normal Controls, UCC= Urothelial Cell Carcinoma. **B**. Scatter plots showing promoter methylation status of *CCNA1, CCND2*, and *CALCA* genes in different grade and stages of UCC. A high percentage of LGUCC can be determined by each of the gene tested.

We tested the promoter methylation of *CCND2, CCNA1* and *CALCA* in urine sediment DNA from primary UCC cases and subjects without any neoplastic disease (controls/normals). By establishing an empiric cutoff, we found *CCND2, CCNA1*, and *CALCA* to be significantly more methylated in urine of UCC patients than controls (Table 2B). The methylation frequency of *CCND2, CCNA1*, and *CALCA* were 38/148 (25.6%), 50/73 (68.4%), and 94/148 (63.5%) respectively for UCC, while 0/56 (0%), 10/60 (16.6%), and 16/56 (28.5%) respectively for controls, (Table 2B). Scatter plots of *CCND2, CCNA1*, and *CALCA* are shown in Figure 2A for UCC cases (148 for CCND2, 73 for CCNA1 and 148 for CALCA) and controls (56 for CCND2, 60 for CCNA1 and 56 for CALCA). Figure 2B shows methylation frequency of *CCNA1, CALCA*, and *CCND2* in different grades and stages of urines from UCC cases. When compared to the current standard method urine cytology (sensitivity of 50% in our cases (44/88), similar to the literature), the sensitivity is higher for any one of the 3 genes methylated (either: *CCND2, CALCA*, and/or *CCNA1*) 72.7% [64/88], with a specificity of 70%. Interestingly, 83% (25/30) of cytology negative LGPUCC cases were positive for one or more of the three methylation markers tested in urine. Out of 101 LGUCC cases, cytology data was available for 70 cases. Detailed information on the methylation and cytology test results of these 70 cases is available in Table 3. The available clinicopathological information for all the 101 LGUCC cases is shown in Supplementary Table 1.

**Table 3:** Clinicopathological and molecular characteristics of urine samples from LGUCC patients tested

Study identification	Cytology	Cystoscopy	Recurrence	Grade	CCNA1	CCND2	CALCA	Any positive
1	+	+	−	LGUCC	NA	−	−	−
2	+	+	−	LGUCC	NA	−	−	−
3	+	+	−	LGUCC	NA	−	−	−
4	+	−	−	LGUCC	NA	−	−	−
5	+	NA	−	LGUCC	NA	−	+	+
6	+	−	−	LGUCC	NA	−	+	+
7	+	+	+	LGUCC	NA	−	+	+
8	+	+	−	LGUCC	NA	−	−	−
9	+	NA	−	LGUCC	NA	−	−	−
10	+	−	NA	LGUCC	NA	+	+	+
11	+	+	−	LGUCC	NA	+	+	+
12	+	+	−	LGUCC	NA	+	+	+
13	+	NA	−	LGUCC	NA	−	+	+
14	+	+	NA	LGUCC	NA	−	−	−
15	+	+	+	LGUCC	−	−	+	+
16	+	+	+	LGUCC	NA	−	−	−
17	+	+	−	LGUCC	−	+	+	+
18	+	+	−	LGUCC	+	+	+	+
19	+	+	−	LGUCC	+	−	−	+
20	+	+	−	LGUCC	+	+	+	+
21	+	−	−	LGUCC	−	+	+	+
22	+	+	−	LGUCC	+	−	−	+
23	+	+	−	LGUCC	+	−	+	+
24	+	+	−	LGUCC	−	−	+	+
25	+	+	−	LGUCC	−	+	+	+
26	+	+	+	LGUCC	−	−	+	+
27	+	+	−	LGUCC	−	−	−	−
28	+	+	−	LGUCC	+	−	+	+
29	+	+	−	LGUCC	+	+	+	+
30	+	+	+	LGUCC	NA	−	−	−
31	+	+	−	LGUCC	+	−	+	+
32	+	+	−	LGUCC	+	−	+	+
33	+	−	NA	LGUCC	+	+	+	+
34	+	+	−	LGUCC	−	−	+	+
35	+	+	−	LGUCC	−	+	+	+
36	+	+	−	LGUCC	−	+	+	+
37	+	+	−	LGUCC	−	+	−	+
38	+	+	−	LGUCC	−	+	+	+
39	+	+	−	LGUCC	−	−	+	+
40	+	+	−	LGUCC	−	+	+	+
41*	−	−	−	LGUCC	−	−	+	+
42*	−	+	+	LGUCC	−	−	+	+
43	−	+	−	LGUCC	−	−	−	−
44*	−	+	−	LGUCC	−	−	+	+
45*	−	+	+	LGUCC	−	−	+	+
46*	−	+	+	LGUCC	−	−	+	+
47*	−	+	NA	LGUCC	−	−	+	+
48*	−	−	−	LGUCC	−	−	+	+
49*	−	+	−	LGUCC	+	−	+	+
50	−	+	+	LGUCC	−	−	−	−
51	−	+	−	LGUCC	−	−	−	−
52*	−	+	−	LGUCC	+	−	+	+
53*	−	+	−	LGUCC	+	−	−	+
54*	−	−	+	LGUCC	+	−	+	+
55*	−	+	+	LGUCC	+	+	+	+
56*	−	+	+	LGUCC	+	−	+	+
57	−	+	−	LGUCC	−	−	−	−
58*	−	+	−	LGUCC	+	+	+	+
59	−	+	−	LGUCC	−	−	−	−
60*	−	+	−	LGUCC	+	+	+	+
61*	−	+	−	LGUCC	+	−	+	+
62*	−	+	−	LGUCC	+	−	+	+
63*	−	+	−	LGUCC	+	−	+	+
64*	−	+	−	LGUCC	−	−	+	+
65*	−	+	−	LGUCC	−	−	+	+
66*	−	+	−	LGUCC	+	+	+	+
67*	−	+	−	LGUCC	NA	−	+	+
68*	−	+	−	LGUCC	NA	+	+	+
69*	−	+	−	LGUCC	NA	+	+	+
70*	−	+	−	LGUCC	NA	+	+	+

* Cytology negative but promoter methylation positive

NA, sample was not available for testing

### Re-expression of *CCNA1* and *CCND2* after 5-aza-deoxycytidine (5-aza-dc) and trichostatin A (TSA) treatment

To determine promoter methylation specific gene silencing, we and others have previously reported pharmacological unmasking strategies for numerous genes in several cell lines of different cancer types [27-29]. Here, to determine whether promoter methylation is inversely correlated with expression of selected genes (*CCNA1* and *CCND2*) from our 8 gene panel, we treated 5 UCC cell lines with 5-aza-dc alone or in combination with TSA (a histone deacetylase inhibitor). Out of the 4 genes associated with recurrence, we tested 2 genes for reactivation after treatment with epigenetic agents. Our findings as a proof of principle indeed showed that *CCND2* and *CCNA1* can be re-expressed with the treatment of epigenetic drugs. Other two genes that showed association with recurrence were previously reported to be re-expressed after treatment with epigenetic drugs ([30, 31]).

**Figure 3:**
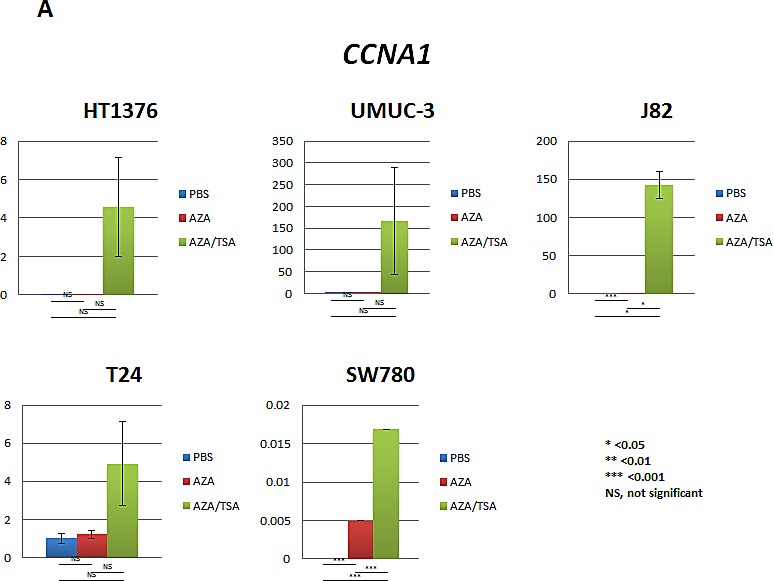

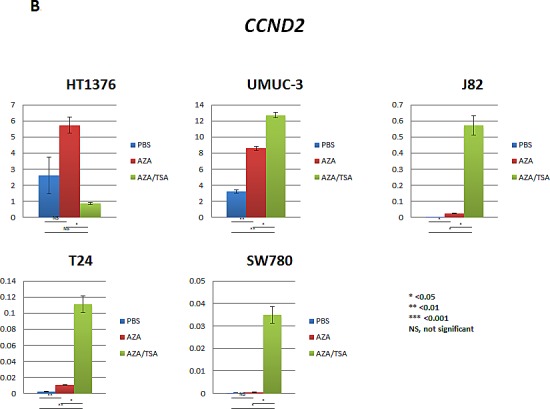
Re-expression of *CCNA1* and *CCNA2* after 5-aza-dc (AZA) and/or TSA treatment of urothelial cancer (UCC) cell lines analyzed by real-time RT-PCR A. Reactivation of *CCNA1* was observed in SW780 and J82 UCC cell lines after 5-aza-dc treatment (*p<0.001*), while robust overexpression of *CCNA1* was observed after combination treatment (*p<0.05*). B. Reactivation of *CCND2* was observed in UMUC-3, J82 and T24 UCC cell lines after 5-aza-dc treatment (*p<0.05*). When using combination treatment with 5-aza and TSA, an increased expression was observed in UMUC-3, J82, T24 and SW780 cell lines (*p<0.05*). In HT1376 cell line, overexpression was observed after 5-aza-dc treatment only (not significant), however, *CCND2* expression noticeably decreased after combination treatment of 5-aza-dc and TSA treatment. PBS was used as treatment control. PBS, phosphate buffered saline; AZA, 5-aza-dc; TSA, trichostatin-A; AZA/TSA, combination treatment with 5-aza-dc and trichostatin-A; NS, not significant; *, *p<0.05*; **, *p<0.01*; ***, *p<0.0001*. t-student test *p* values.

Two UCC cell lines (SW780 and J82) showed re-expression of *CCNA1* after 5-aza-dc treatment (*p < 0.001*) and after combination treatment (*p < 0.05* in J82 and *p < 0.001* in SW780) (Figure 3A). *CCND2* showed a similar pattern of re-expression with 5-aza-dc treatment (UMUC-3, J82 and T24) and after combination treatment (UMUC-3, J82, T24 and SW780). CCND2 expression was down-regulated only in the HT1376 cell line after treatment with 5-aza-dc and trichostatin-A (Figure 3B). To determine whether promoter methylation of *CCNA1* and *CCND2* are inversely correlated with expression, we performed QMSP assay for *CCNA1* and *CCND2*. Among the 5 UCC cell lines, promoter methylation of *CCNA1* is inversely correlated with expression in J82 and SW780 (data not shown). Similarly, for *CCND2*, we observed that promoter methylation is inversely correlated with expression in J82, SW780 and T24 cell lines (data not shown). These findings suggest that both DNA methylation and histone deacetylation play a role in *CCND2* and *CCNA1* genes silencing.

### *CCNA1* suppresses proliferation and colony formation of UCC cells

**Figure 4:**
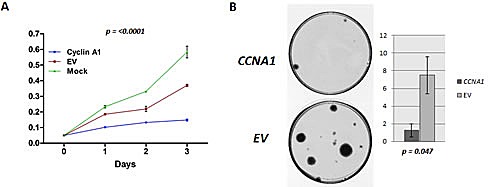
Ectopic expression of *CCNA1* inhibits tumor cell growth **A**. The MTT assay was performed in a J82 cell line transiently transfected with *pCMS-EGFP-cyclinA1* and empty *pCMS-EGFP* plasmid (control). Forceful expression of *CCNA1* significantly decreased the viable cells in comparison with empty vector (EV) control and cells without any transfection (Mock) (p=<0.0001) **B**. The effect of ectopic *CCNA1*-expression on bladder carcinoma cell clonogenicity was investigated by colony formation assay. J82 cells were transfected with *pCMS-EGFP-cyclinA1* and empty *pCMS-EGFP* plasmid (control). Left panel, images of the colony formation assays. Right panel, Bar graph representing the number of colonies observed (larger than 2mm). Significantly fewer numbers of colonies were observed after over expressing *CCNA1* containing vector in J82 cells (p=<0.047).

To evaluate the effect of *CCNA1* on the growth of UCC cell lines, *CCNA1* was forcefully expressed in J82 cell line. Verification of *CCNA1* overexpression was done by Q-RT-PCR and immunoblotting analysis 48h after transfection (data not shown). As shown in Figure 4A, forced expression of *CCNA1* significantly inhibited growth of J82 cells in culture (p=<0.0001), where cell growth inhibition is mediated in a time-dependent manner. To assess long-term growth, colony focus assays were performed after treatment of *CCNA1* transfected cells with the plasmid selection marker G418 for 2 weeks. *CCNA1* showed potent tumor suppressive activity by markedly reducing the colony-forming ability of the cells as shown in Figure 4B.

## DISCUSSION

The main goal of this study was to evaluate wheter the status of promoter methylation of a candidate gene or gene-panel was different among LGPUCC that recurred and those that did not. For further monitoring of patients after TURBT of LGPUCC, a non-invasive screening test is essential in order to avoid invasive and costly procedures such as cystoscopy. To this end, we evaluated the feasibility of a set of genes that predicts recurrence in primary LGPUCC for the non-invasive detection of UCC in urine sediments. To elucidate the biologic relationship of *CCNA1* silencing in the context of UCC, we performed different *in vitro* assays and our data is consistent with our findings in human primary LGPUCC that *CCNA1* is a potential tumor suppressor gene.

We analyzed promoter methylation of 8 genes (*ARF, TIMP3, RAR-β2, NID2, CCNA1, AIM1, CALCA* and *CCND2*) in the recurrent and non-recurrent LGPUCC and observed that the methylation frequencies of 3 genes (*NID2, CCNA1*, and *CCND2*) were significantly higher in recurrent LGPUCC. The frequency of promoter methylation of *CALCA* was borderline significant (p=0.09). We had previously shown a UCC specific methylation pattern for *CCND2, CCNA1* and *CALCA* [11]. In the latter study, we analyzed 93 UCC samples and 26 normal uro-epithelium samples and observed 57% of methylation in *CCNA1* in tumors while no methylation was observed in controls, 57% in *CCND2* in tumors while 19% in normals, and 65% in *CALCA* with 15% in normal uro-epithelium [11]. *AIM1*, a gene without a clear functional data, showed a UCC specific pattern (over 70% in UCC) in our previous study [11], however, although we found high frequency of methylation in the tested primary LGPUCC samples in this study [22/36(61%)], *AIM1* was not differentially methylated among recurrent and non-recurrent LGPUCC. This could be due to small sample size in that study or *AIM1* inactivation may be related to both initiation and progression of UCC. Ulazzi et al., [30] were the first group to demonstrate *NID2* methylation in a cancer specific manner, in human gastrointestinal cancer; promoter hypermethylation of *NID2* was shown in 14 out of 48 colon carcinoma samples analyzed compared to 0/24 normal colon, 19/20 of the gastric carcinomas, and 0/13 normal gastric mucosa. Moreover, Renard et al. [14] performed a pharmacologic unmasking method in four UCC cell lines, generated a list of candidate methylated genes, and subsequently performed methylation-specific PCR (MSP) in UCC and normal tissue samples. In their study, *NID2* showed methylation in 66 out of 91 UCC tissues and 0 out of 39 normal urothelial tissues analyzed. They then analyzed promoter methylation of *NID2* and *TWIST1* as a panel in urine DNA from UCC patients and controls. This two gene panel detected UCC patients with 90% sensitivity and 93% specificity while the sensitivity and specificity of cytology test in the same cohort were 48% and 96% respectively. When analyzing only LGPUCC, they observed a sensitivity of 80% (training set) and 89% (validation set) compared to 45% and 44% from cytology, with a sensitivity of 94% and 91% compared to cytology's sensitivity of 97% and 95%. In our cohort, cytology data was available for 70 LGPUCC cases, and the cytology sensitivity for LGPUCC was 50%, while the methylation sensitivity was about 79% using our 3 gene panel (methylation in either: *CCND2, CALCA*, and/or *CCNA1*), values comparable to the 2 gene panel showed by Renard et al.'s study. It would be interesting to analyze a cohort of urine samples from LGUCC cases for all the 5 genes (*CCNA1, CCND2, CALCA, NID2 and TWIST1)* and determine the sensitivity and specificity of the test. A prospective study using appropriate controls and number of samples is necessary to determine the clinical utility of these markers. Furthermore, subsequently collected urine samples in follow-up visits need to be tested to determine the marker's usefulness in reducing cystoscopy in follow-up visits.

In our study, we considered any recurrence as presence of recurrence. Due to the limited number of primary LGPUCC samples we were not able to stratify the cases based on length of follow-up time to recurrence. Several studies previously used a minimum follow-up period of 12 months after TURBT to evaluate potential biomarkers at the time of diagnosis for the prediction of recurrence. However, in our study, using 12 months as the cutoff, we ended up with only 12 non-recurrent and 13 recurrent LGPUCC cases for analysis, which is not enough for meaningful statistical analysis. Further studies using larger cohort are necessary. We are now following-up our cohorts in order to better delineate the precise role of a given marker in relation to recurrence.

The ultimate goal of this pilot study was to identify markers that could be detected in urine samples from LGPUCC patients obtained during follow-up visits after TURBT in order to reduce the need of performing cystoscopies. An optimal non-invasive molecular test will allow for screening of patients before an invasive procedure, which might also reduce the number of cystoscopies necessary in surveillance of non–muscle-invasive bladder cancer. If the test has high sensitivity and specificity, cystoscopy would only be performed in patients who are positive for the non-invasive test. We are in the process of longitudinally collecting follow-up urine samples in subsequent visits of LGPUCC patients following cystoscopy and TURBT. Urine DNA methylation testing of such samples needs to be performed to evaluate the utility of such a test potentially substituting for and therefore extending the currently adopted interim follow-up scheme between cystoscopies.

Given the lack of an adequate number of sequentially collected urine samples, in the current cohort, we focused on determining the feasibility of the detection of cancer specific methylation of three genes by testing urine from UCC cases and controls. These 3 genes (*CCND2, CCNA1* and *CALCA*) were selected from our panel of 8 genes that were analyzed in non-recurrent and recurrent primary LGPUCC. Our findings support that the presence of cancer can be determined by testing the promoter methylation of these genes with high specificity in urine. To our best knowledge, these 3 genes had not been tested previously in LGPUCC urine samples by our group and others; and can be incorporated in a gene panel for future early detection and monitoring of LGPUCC patients. We analyzed 148 urine samples, and of the 125 with known grade, 101 of those urine samples were collected from LGPUCC patients. 97 of 101 LGPUCC cases were methylation positive for at least one of the 3 markers tested. Interestingly, our methylation assays were able to detect 25 LGPUCC cases where urine cytology was negative. The latter suggests that these markers may have potential for non-invasive monitoring of LGPUCC after TURBT. Due to the limited amount of bisulfite converted DNA, we were not able to assess *NID2* methylation in urine DNA of UCC cases and controls. However, this gene has previously shown excellent discrimination between urine of UCC patients and controls, with a sensitivity of 94% and a specificity of 91% [14].

We tested the relevance of promoter methylation compared to expression of two members (*CCNA1* and *CCND2*) of the cyclin family in this study and in general methylation was correlated with expression in UCC cell lines. *CCNA1* is known to be a downstream target of TP53 [32], and *CCNA1* methylation was shown to be inversely related to p53 mutational status in primary Head and Neck Squamous cell carcinomas (HNSCC). Forced expression of *CCNA1* resulted in robust induction of wild-type p53 in HNSCC cell lines [16]. *CCNA1* is frequently inactivated in UCC [11], which indicates its anti-proliferative activity; however, in a recent study, it has been implicated that *CCNA1* contributes to prostate cancer invasion and metastasis [33]. It may be speculated that *CCNA1* may play different roles in different tumor types and in different biological contexts. Our data in non-recurrent and recurrent primary LGPUCC demonstrated that *CCNA1* is significantly more methylated (e.g. silenced) in recurrent LGPUCC than in non-recurrent LGPUCC. We speculate that inactivation of *CCNA1* may have some role in recurrence; although, we do not have definite functional evidence. However, limited functional studies performed in this study for *CCNA1* are in the same direction as our findings in primary LGPUCC, which is that it has growth suppressive activity. Further studies need to be performed to understand the mechanistic role of *CCNA1* in the pathogenesis and recurrence of LGPUCC.

Although our limited data suggests that *CCND2* is a potential tumor suppressor gene (TSG) in UCC, the role of *CCND2* in human cancer is controversial. It has been proposed as a proto-oncogene, and its overexpression has been reported in gastric cancer [21], ovarian and testicular tumors [34], and hematopoietic cell cancer [35, 36]. In contrast, reduction or lack of *CCND2* expression has also been reported in gastric cancer [20], breast cancer [37, 38], prostate cancer [39], and lung cancer [18], suggesting that *CCND2* may function as a TSG. Recently, transcriptional silencing by aberrant methylation of promoter region of the *CCND2* gene has been found in gastric cancer [20], breast cancer [38, 40], prostate cancer [39, 41], lung cancer [18], and Epstein–Barr virus-positive Burkitt's lymphoma [42]. These previous reports suggest that *CCND2* may function as an oncogene or a TSG, and the critical biological role of this molecule needs to be explored in the biological context of UCC pathogenesis. A recent study [43] reported that reduced expression of *CCND2* in stage III non-small cell lung cancer (NSCLC) is associated with poor recurrence-free survival. In the present study, we found that *CCND2* is significantly more methylated in recurrent than in non-recurrent LGPUCC, which indicates a potential similar biological role for CCND2 in NSCLC and LGPUCC, two types of cancers related to smoking.

All of the remaining studied genes have been previously described as hypermethylated in UCC: *CALCA* (calcitonin-related polypeptide alpha is involved in calcium regulation and acts to regulate phosphorus metabolism) was not only shown to have a UCC specific methylation pattern, but was also correlated to later stage tumors (>pT2) [11]. *ARF* or *p14*, an important player in cell cycle regulation, has been previously studied in UCC, and the range of methylation frequency observed was between 0 and 56% [44, 45]. Dominguez et al. [45] showed that the presence of *p14* methylation in the plasma was significantly associated to recurrence in UCC. In our cohort, we could not confirm this data in tumor samples, which may be due to the limited sample size. *RAR-β2*, involved in cell differentiation, has been analyzed in UCC to give diverse results, from 2 to almost 90% methylation [46, 47]. Promoter methylation of *TIMP3* (tissue inhibitor of metalloproteinases-3) in urine DNA was shown to be an independent prognostic factor for UCC [13]; however, here, we did not observe a correlation with recurrence in primary LGPUCC samples. An extended study using a larger primary LGPUCC cohort will elucidate the role of *TIMP3* in recurrence of LGPUCC.

Although our group and others have shown evidence that some methylation markers have potential for noninvasive UCC detection and for predicting patient survival and tumor progression [12, 13, 47], there are still no methylation based markers implemented in the clinical practice. For the prediction of recurrence of LGPUCC, Tada et al. [48] reported that hypermethylation of death-associated protein kinase (*DAPK1*) might be a useful prognostic marker for disease recurrence in superficial UCC. In their study, a total of 88% of papillary UCC with *DAPK1* methylation recurred within 15 months, while 71% of tumors that were not methylated for *DAPK1* had not recurred within 24 months. Nevertheless, previous studies have shown infrequent *DAPK1* methylation in UCC samples [49, 50].

In summary, this work not only sheds light onto new potential methylation based markers associated with recurrent LGPUCC, but also shows the potential of detection of 3 novel genes in urine sediments and demonstrates initial evidence of tumor suppressive activities of *CCNA1* in the context of the biology of UCC cell lines. A larger prospective study with longitudinal follow-up with an independent cohort is needed to assess and validate our preliminary promising findings.

## MATERIALS AND METHODS

### Tissue and urine samples

A total of 36 formalin-fixed paraffin-embedded (FFPE) primary LGPUCC tissues were obtained from patients who underwent therapeutic surgery at The Johns Hopkins Hospital. The demographic and clinical information was obtained from the computerized tumor registry at The Johns Hopkins Healthcare System. Among the 36 LGPUCC samples, 17 were collected from patients who did not recur during any follow-up periods, and the remaining 19 were primary tumor samples that recurred within the follow-up periods after TURBT. We also performed analysis by considering the follow-up periods of 12, 18, and 24 months for recurrence to observe the association with promoter methylation of candidate markers. To be included in the cohort, an eligible patient had to have a confirmed diagnosis of LGPUCC and a sufficient amount of archived tumor material for DNA extraction. A detailed summary of all the LGPUCC samples with their clinico-pathological parameters is available in Table 1.

To determine the feasibility of detecting promoter methylation of genes in urine related to LGPUCC recurrence, we tested promoter methylation of 3 genes (*CCND2, CCNA1* and *CALCA)* in the urine sediment of 73 to 148 patients with primary UCC (101 LGPUCC, 24 high grade UCC and 23 unknown grade) and of 56 to 60 healthy subjects without any known neoplastic diseases. Fifty milliliters of voided urine were collected from all cases prior to definite surgery. Urine samples were spun at 3000 × g for 10 min and washed twice with phosphate-buffered saline. All samples were stored at −80°C.

Approval for research on human subjects was obtained from The Johns Hopkins University institutional review boards. This study qualified for exemption under the U.S. Department of Health and Human Services policy for protection of human subjects [45 CFR 46.101(b)].

### Cell lines

All of the cell lines (HT1736, T24, J82, UM-UC-3 and SW780) used in this study were cultured accordingly to the recommendations of the American Type Culture Collection (ATCC) (Manassas, VA, USA), from where they were purchased.

### DNA extraction

All original LGPUCC histologic slides were reviewed to reconfirm the diagnosis by a senior urologic pathologist (GN). A representative formalin-fixed paraffin-embedded (FFPE) block that contained sufficient amount of tissue was retrieved for DNA extraction and several 10 micron slides were obtained from each block. The presence of tumor cells was confirmed by staining the first and last slides of the representative block with hematoxylin & eosin. The tumor samples were microdissected to obtain >70% of neoplastic cells. DNA from tumors, cell lines and urine sediments were extracted using the phenol-chloroform extraction protocol followed by ethanol precipitation as described previously [51].

### Bisulfite Treatment

DNA extracted from primary tumors, cell lines and urines was subjected to bisulfite treatment, which converts unmethylated cytosine residues to uracil residues, as described previously [52]. EpiTect Bisulfite kit (Cat No. 59104, from QIAGEN Inc. Valencia, CA – 91355) was used for this conversion, following the manufacturer's instructions.

### Quantitative fluorogenic methylation specific PCR (QMSP)

Bisulfite-modified DNA was used as a template for fluorescence-based real-time PCR, as previously described [12]. Amplification reactions were carried out in triplicate in a final volume of 20 μL that contained 2 μL of bisulfite-modified DNA; 600 nM concentrations of forward and reverse primers; 200 nM probe; 0.6 U of platinum Taq polymerase (Invitrogen, Frederick, MD); 200 μM concentrations each of dATP, dCTP, dGTP and dTTP; and 6.7 mM MgCl_2_. Primers and probes were designed to specifically amplify the promoter region of *ARF, TIMP3, RAR-β2, CCNA1, NID2, AIM1, CALCA, CCND2*, and of a reference gene, *β-actin*; primer and probe sequences and annealing temperatures are provided in Supplemental Table 2A. Amplifications were carried out in 384-well plates in a 7900HT sequence detector (Applied Biosystems, Foster City, CA) using the following conditions: 95 °C for 3 minutes, followed by 50 cycles at 95 °C for 15 seconds and 60 °C for 1 minute. Results were analyzed by a sequence detector system (SDS 2.4; Applied Biosystems). Each plate included patient DNA samples, and positive and negative controls. Serial dilutions (90–0.009ng) of *in vitro* methylated DNA were used to construct a calibration curve for each plate. All samples were within the assay's range of sensitivity and reproducibility based on amplification of internal reference standard (threshold cycle [CT] value for *β-actin* of 40). The relative level of methylated DNA for each gene in each sample was determined as a ratio of methylation specific PCR-amplified gene to *β-actin* (reference gene) and then multiplied by 1000 for easier tabulation (average value of triplicates of gene of interest divided by the average value of triplicates of *β-actin* × 1000). The presence or absence of methylation was compared between recurrent and non-recurrent groups using cross-tabulations and χ^2^ or Fisher's exact tests as appropriate. The cutoff value for each gene was established by maximizing sensitivity and specificity. We determined the empiric cutoff on individual ROC (receiver operating curves) that makes optimal differences between the two groups (maximizing sensitivity and specificity). In our previous study [12], we found that dichotomization and logistic regression essentially produces similar results. Furthermore, considering the small number of sample size, we decided to use empiric cutoffs to see the differences between the two groups.

### 5-aza-deoxycytidine (5-aza-dc) and Trichostatin A (TSA) treatment

UCC cells were seeded in 75 cm^2^ culture flasks at a density of 2 × 10^5^ and incubated at 37ºC in 5% CO2/95% air overnight. Cells were then treated with 5μM of 5-aza-dc (Sigma Chemical, Sigma, USA) for 5 days. Medium with 5-aza-dc was changed daily. Additionally, combination treatment with 5-aza-dc and TSA was performed by adding 5μM of 5-aza-dc daily for 5 days and TSA (300 nmol/L; Sigma) was added to the medium for the final 24 hours. Cells were harvested after the last day of treatment (5-aza-dc only and 5 aza-dc + TSA) for RNA extraction and the analysis of gene expression were performed by Quantitative Reverse Transcriptase-PCR (Q-RT-PCR). PBS (phosphate buffered saline) alone was used as a control to exclude non-specific solvent effects on cells. All experiments were run independently twice.

### RNA extraction, cDNA synthesis and Quantitative Reverse Transcription-PCR (Q-RT-PCR)

RNA was extracted using Qiazol Lysis reagent (Qiagen, Valencia, CA) according to the manufacturer's instructions. One microgram of total RNA was used for cDNA conversion using the Quantitect Reverse Transcription Kit (Qiagen, Valencia, CA), following manufacturer's protocol.

Q-RT-PCR was performed using the SYBR Green chemistry in a 7900HT Real-Time PCR System (Applied Biosystems, Foster City, CA). The reaction mixture contained 2.6 μl of DEPC-treated water, 5 μl Power SYBR Green PCR Master Mix (Applied Biosystems), and 0.2 μl of gene-specific primers (final concentration, 50 nM each), in a final reaction volume of 10 μl. The RT-PCR primer sequences are available in Supplementary Table 2B. The cycling conditions were as follows: a denaturation step at 95ºC for 3 min, followed by 40 cycles of 95ºC for 15 s, 60ºC for 60 s, and a final step for the generation of a dissociation curve to distinguish between the main RT-PCR product and primer-dimers. Calculations were made with the use of the comparative CT (2_ΔΔCT) method. GAPDH was used as an internal control gene to normalize the reaction for the amount of RNA added to the reverse transcription reactions [53]. Each real-time PCR reaction was performed in triplicates to evaluate the reproducibility of data.

### Cellular Viability Assay (MTT Assay)

Cellular proliferation was measured by the thiazolyl blue tetrazolium bromide (MTT) (Sigma-Aldrich) according to the manufacturer's instructions. Briefly, J82 cells were counted and seeded at a density of 1000 cells per well on 96 well plates, in triplicates. The cells were allowed to attach overnight. One plate of cells was seeded in the absence of serum to synchronize growth, while another plate was seeded in the presence of serum (10% FBS). Transfection with the *pCMS-EGFP-cyclinA1* and *pCMS-EGFP-MOCK* (control) vectors (kindly provided by Dr. Jenny L. Persson, Clinical Research Center, Malmo, Sweden) was performed using Fugene HD transfection reagent (Roche). The cell doubling time was calculated during exponential growth phase (0, 24, 48 and 72 hrs). Ten microliters of MTT labeling reagent (5 mg/mL MTT) were added to the culture media without fetal bovine serum (FBS), which was then incubated in the dark for additional 3h at 37ºC. This step was followed by cell lysis with the addition of 100μL DMSO. Spectrophotometric readings (A_570 nm_ to A_650 nm_) were obtained on a Spectra Max 250 96-well plate reader (Molecular Devices). Each assay was carried out in triplicate and each experiment was repeated at least two times.

### Transfection and colony formation assay

Colony formation assays were performed in monolayer culture [54]. J82 cells were plated at a density of 2 × 10^4^ cells/well using 6-well plates, and transfected with 1 μg of either the *pCMS-EGFP-cyclinA1* or *pCMS-EGFP-MOCK* (control) vectors using Fugene HD transfection reagent (Roche), according to the manufacturer's protocol. The cells were then detached and plated on 100 mm tissue culture dishes at 24 to 48 hrs post-transfection and simultaneously harvested at 48 hr after transfection to confirm the overexpression of *CCNA1* at the mRNA level (Q-RT-PCR) and protein level. Cells were cultured for 2 weeks in medium containing 400 μg/mL of G418 (Cellgro, Manassas, VA). The cultures were washed twice with phosphate buffered saline (PBS), fixed with 25% acetic acid and 75% methanol at room temperature for 10 minutes, and then stained with 0.1% crystal violet. Colonies were counted and the number of colonies per dish was averaged from three independent experiments that were performed. This colony formation assay was repeated three independent times.

### Statistical Analysis

The presence or absence of methylation was compared between the groups (recurrent and non-recurrent UCC; and urine of UCC cases and controls) using cross-tabulations and χ^2^ or Fisher's exact tests as appropriate. Student t-test was used to compare the averages of duplicates or triplicates among the re-expression experiments, cell viability and colony formation assays.

## SUPPLEMENTARY MATERIAL AND TABLES


