# Supplementary material for: An epigenetic marker panel for recurrence risk prediction of low grade papillary urothelial cell carcinoma (LGPUCC) and its potential use for surveillance after transurethral resection using urine
Source: Oncotarget. 2014 Jun 23;5(14):5218–33. doi: 10.18632/oncotarget.2129 (PMC4170626; doi:10.18632/oncotarget.2129)
Supplement: Supplementary file 1 [file oncotarget-05-5218-s001.pdf]

An epigenetic marker panel for recurrence risk prediction of low grade papillary urothelial cell carcinoma (LGPUCC) and its potential use for surveillance after transurethral resection using urine

Supplementary Material

Sup Table 1: Clinicopathological and molecular characteristics of urine samples from UCC patients tested

Study identification	Cytology	Cystoscopy	Recurrence	Grade	*CCNA1*	*CCND2*	*CALCA*	Any positive
1	+	+	-	LGUCC	NA	-	-	-
2	+	+	-	LGUCC	NA	-	-	-
3	+	+	-	LGUCC	NA	-	-	-
4	+	-	-	LGUCC	NA	-	-	-
5	+	NA	-	LGUCC	NA	-	+	+
6	+	-	-	LGUCC	NA	-	+	+
7	+	+	+	LGUCC	NA	-	+	+
8	+	+	-	LGUCC	NA	-	-	-
9	+	NA	-	LGUCC	NA	-	-	-
10	+	-	NA	LGUCC	NA	+	+	+
11	+	+	-	LGUCC	NA	+	+	+
12	+	+	-	LGUCC	NA	+	+	+
13	+	NA	-	LGUCC	NA	-	+	+
14	+	+	NA	LGUCC	NA	-	-	-
15	+	+	+	LGUCC	-	-	+	+
16	+	+	+	LGUCC	NA	-	-	-
17	+	+	-	LGUCC	-	+	+	+
18	+	+	-	LGUCC	+	+	+	+
19	+	+	-	LGUCC	+	-	-	+
20	+	+	-	LGUCC	+	+	+	+
21	+	-	-	LGUCC	-	+	+	+
22	+	+	-	LGUCC	+	-	-	+
23	+	+	-	LGUCC	+	-	+	+
24	+	+	-	LGUCC	-	-	+	+
25	+	+	-	LGUCC	-	+	+	+
26	+	+	+	LGUCC	-	-	+	+
27	+	+	-	LGUCC	-	-	-	-
28	+	+	-	LGUCC	+	-	+	+
29	+	+	-	LGUCC	+	+	+	+

30	+	+	+	LGUCC	NA	-	-	-
31	+	+	-	LGUCC	+	-	+	+
32	+	+	-	LGUCC	+	-	+	+
33	+	-	NA	LGUCC	+	+	+	+
34	+	+	-	LGUCC	-	-	+	+
35	+	+	-	LGUCC	-	+	+	+
36	+	+	-	LGUCC	-	+	+	+
37	+	+	-	LGUCC	-	+	-	+
38	+	+	-	LGUCC	-	+	+	+
39	+	+	-	LGUCC	-	-	+	+
40	+	+	-	LGUCC	-	+	+	+
41	-	-	-	LGUCC	-	-	+	+
42	-	+	+	LGUCC	-	-	+	+
43	-	+	-	LGUCC	-	-	-	-
44	-	+	-	LGUCC	-	-	+	+
45	-	+	+	LGUCC	-	-	+	+
46	-	+	+	LGUCC	-	-	+	+
47	-	+	NA	LGUCC	-	-	+	+
48	-	-	-	LGUCC	-	-	+	+
49	-	+	-	LGUCC	+	-	+	+
50	-	+	+	LGUCC	-	-	-	-
51	-	+	-	LGUCC	-	-	-	-
52	-	+	-	LGUCC	+	-	+	+
53	-	+	-	LGUCC	+	-	-	+
54	-	-	+	LGUCC	+	-	+	+
55	-	+	+	LGUCC	+	+	+	+
56	-	+	+	LGUCC	+	-	+	+
57	-	+	-	LGUCC	-	-	-	-
58	-	+	-	LGUCC	+	+	+	+
59	-	+	-	LGUCC	-	-	-	-
60	-	+	-	LGUCC	+	+	+	+
61	-	+	-	LGUCC	+	-	+	+
62	-	+	-	LGUCC	+	-	+	+
63	-	+	-	LGUCC	+	-	+	+
64	-	+	-	LGUCC	-	-	+	+
65	-	+	-	LGUCC	-	-	+	+
66	-	+	-	LGUCC	+	+	+	+
67	-	+	-	LGUCC	NA	-	+	+
68	-	+	-	LGUCC	NA	+	+	+
69	-	+	-	LGUCC	NA	+	+	+
70	-	+	-	LGUCC	NA	+	+	+

71	NA	NA	NA	NA	NA	-	+	+
72	NA	+	NA	LGUCC	NA	-	-	-
73	NA	NA	NA	NA	NA	-	-	-
74	NA	+	-	LGUCC	NA	+	+	+
75	NA	+	+	HGUCC	NA	-	-	-
76	-	-	+	HGUCC	NA	-	-	-
77	-	-	+	HGUCC	NA	-	-	-
78	NA	NA	NA	NA	NA	-	+	+
79	NA	NA	NA	NA	NA	-	+	+
80	NA	NA	NA	NA	NA	-	-	-
81	NA	NA	NA	NA	NA	+	+	+
82	-	-	-	NA	NA	-	+	+
83	NA	NA	NA	NA	NA	-	+	+
84	NA	NA	NA	NA	NA	-	+	+
85	NA	NA	NA	NA	NA	-	-	-
86	NA	NA	NA	NA	NA	-	-	-
87	-	+	+	HGUCC	NA	-	-	-
88	NA	+	-	LGUCC	+	+	-	+
89	-	+	+	HGUCC	+	+	-	-
90	NA	NA	NA	NA	-	-	-	-
91	+	-	+	HGUCC	-	-	-	-
92	NA	NA	NA	HGUCC	NA	-	-	-
93	NA	NA	NA	LGUCC	NA	-	+	+
94	NA	NA	NA	NA	NA	-	-	-
95	-	1	1	HGUCC	NA	-	+	+
96	NA	NA	NA	LGUCC	+	-	+	+
97	+	+	-	HGUCC	-	-	-	-
98	NA	NA	NA	NA	+	-	-	+
99	-	+	-	HGUCC	-	-	-	-
100	-	+	-	HGUCC	+	-	-	+
101	NA	NA	NA	HGUCC	-	-	+	+
102	NA	NA	NA	LGUCC	+	-	+	+
103	NA	+	-	LGUCC	-	+	+	+
104	NA	NA	NA	NA	+	-	-	+
105	2	2	2	HGUCC	-	-	-	-
106	NA	NA	NA	LGUCC	-	-	-	-
107	NA	NA	NA	NA	NA	-	+	+
108	NA	NA	NA	NA	NA	-	-	-
109	NA	NA	NA	NA	+	-	+	+
110	-	+	+	HGUCC	-	-	+	+
111	-	+	-	HGUCC	-	-	+	+

112	NA	NA	NA	HGUCC	+	+	+	+
113	-	+	_	HGUCC	-	-	-	-
114	NA	NA	NA	NA	+	-	+	+
115	NA	NA	NA	NA	+	-	-	+
116	NA	+	-	LGUCC	-	-	+	+
117	NA	+	+	LGUCC	-	-	+	+
118	NA	+	-	LGUCC	+	+	+	+
119	NA	+	-	LGUCC	+	-	+	+
120	1	+	+	HGUCC	-	-	-	-
121	NA	-	-	HGUCC	+	-	-	+
122	NA	NA	NA	LGUCC	-	-	-	-
123	NA	-	-	LGUCC	-	+	+	+
124	NA	+	-	LGUCC	NA	+	+	+
125	NA	+	-	LGUCC	NA	-	+	+
126	NA	+	-	HGUCC	+	+	+	+
127	NA	+	-	LGUCC	+	-	+	+
128	NA	-	-	LGUCC	-	+	+	+
129	NA	-	-	HGUCC	-	-	-	-
130	NA	-	-	LGUCC	-	+	+	+
131	NA	NA	NA	HGUCC	-	+	+	+
132	NA	NA	NA	LGUCC	+	+	+	+
133	NA	NA	NA	LGUCC	+	-	-	-
134	NA	+	-	LGUCC	-	-	+	+
135	NA	-	-	LGUCC	NA	-	-	-
136	-	-	-	HGUCC	NA	-	+	+
137	NA	-	-	LGUCC	NA	-	+	+
138	NA	+	-	LGUCC	NA	+	+	+
139	NA	-	-	LGUCC	NA	-	+	+
140	NA	+	-	LGUCC	NA	-	+	+
141	NA	+	-	LGUCC	NA	+	+	+
142	NA	+	-	LGUCC	NA	+	+	+
143	NA	NA		NA	NA	-	-	-
144	+	+	+	HGUCC	NA	-	-	-
145	NA	+	-	HGUCC	NA	-	-	-
146	NA	NA	NA	NA	NA	-	-	-
147	-	+	+	HGUCC	NA	-	-	-
148	NA	+	-	LGUCC	+	-	+	+

NA, sample/data was not available

Supplemental Table 2.

A. Primers and probes sequences and annealing temperatures (T°C) used for QMSP.

Gene	Forward 5´-3´ (primer)	Probe 5´-3´ (6-FAM-5'- 3'-6-TAMRA)	Reverse 5´-3´ (primer)	T°C
βActin	TGGTGATGGAGGAGGTTTAGTAAGT	ACCACCACCCAACACACAATAACAAACACA	AACCAATAAAACCTACTCCTCCCTTAA	60
AIM1	CGCGGGTATTGGATGTTAGT	GGGAGCGTTGCGGATTATTCGTAG	CCGACCCACCTATACGAAAA	60
ARF	ACGGGCGTTTTCGGTAGTT	CGACTCTAAACCCTACGCACGCGAAA	CCGAACCTCCAAAATCTCGA	60
CALCA	GTTTTGGAAGTATGAGGGTGACG	ATTCCGCCAATACACAACAACCAATAAACG	TTCCCGCCGCTATAAATCG	60
CCNA1	TCGCGGCGAGTTTATTCG	CGTTATGGCGATGCGGTTTCGG	CCGACCGCGACAAACG	60
CCND2	TTTGATTTAAGGATGCGTTAGAGTACG	AATCCGCCAACACGATCGACCCTA	ACTTTCTCCCTAAAAACCGACTACG	60
NID2	GCGGTTTTTAAGGAGTTTTATTTTC	ACGCCGCTACCCCAAACCTTACGA	CTACGAAATTCCCTTTACGCT	60
RARβ2	GGGATTAGAATTTTTTATGCGAGTTGT	TGTCGAGAACGCGAGCGATTCG	TACCCCGACGATACCCAAAC	60
TIMP3	GCGTCGGAGGTTAAGGTTGTT	AACTCGCTCGCCCGCCGAA	CTCTCCAAAATTACCGTACGCG	62

B. Primers sequences and annealing temperatures (T°C) used for Quantitative real-time RT-PCR.

Gene	Forward 5´-3´ (primer)	Reverse 5´-3´ (primer)	T°C
GAPDH	CGTCTTCACCACCATGGAGA	CGGCCATCACGCCACAGTTT	58
CCNA1	CTCCTGTCTGGTGGGAGGA	TCAGGTGTTATTCTGGATCAG	58
CCND2	CGCAAGCATGCTCAGACCTT	CCACCGTCGATGATCGCA	58
